# Goal and shot prediction in ball possessions in FIFA Women’s World Cup 2023: a machine learning approach

**DOI:** 10.3389/fpsyg.2025.1516417

**Published:** 2025-01-31

**Authors:** Iyán Iván-Baragaño, Antonio Ardá, José L. Losada, Rubén Maneiro

**Affiliations:** ^1^Department of Sport Sciences, Faculty of Medicine, Health and Sports, Universidad Europea de Madrid, Madrid, Spain; ^2^Department of Physical and Sport Education, University of A Coruña, A Coruña, Spain; ^3^Department of Social Psychology and Quantitative Phycology, University of Barcelona, Barcelona, Spain; ^4^Faculty of Education and Sport, University of Vigo, Vigo, Spain

**Keywords:** female football, women’s soccer, predictive models, machine learning, performance analysis, FIFA Women’s World Cup 2023

## Abstract

**Introduction:**

Research in women’s football and the use of new game analysis tools have developed significantly in recent years. The objectives of this study were to create two predictive classification models to forecast the occurrence of a shot or a goal in the FIFA Women’s World Cup 2023 and to identify the associated technical-tactical indicators to these outcomes.

**Methods:**

A total of 2,346 ball possessions were analyzed using an observational design, mapping two different target variables (Success = Goal and Success2 = Goal or Shot) with a relative frequency of 1.28 and 8.35%, respectively. The predictive capacity was tested using Random Forest and XGBoost and finally and SHAP values were calculated and visualized to understand the influence of the predictors.

**Results:**

Random Forest technique showed greater efficacy, with recall and sensitivity above 93% in the resampled dataset. However, recall on the original test sample was 13% (Success = Shot or Goal) and 0% (Success = Goal), demonstrating the models’ inability to predict rare events in football, such as goals. The indicators with the greatest influence on the outcome of these possessions were related to the possession zone, attack duration, number of passes, and starting zone, among others.

**Conclusion:**

The results highlight the need to incorporate a greater number of predictive variables in the models and underline the difficulty of predicting events such as goals and shots in women’s football.

## 1 Introduction

The analysis of technical-tactical performance in men’s football began to develop significantly in the late 20th and early 21st centuries (Hughes and Bartlett, 2002), using notational and observational records (Preciado et al., 2019). Later, with the use of new technologies, this analysis started to be conducted on data obtained from positional sensors such as Global Positioning System (GPS) and Local Positioning System (LPM) (Low et al., 2020). In the case of women’s football, the lower participation of women in the sport and a lack of social and research interest delayed the publication of the first studies by more than a decade (Kirkendall, 2007; Mara et al., 2012; Leite, 2013). A significant increase in research occurred starting in 2020, coinciding with the FIFA Women’s World Cup 2019 (Lee and Mills, 2021; Iván-Baragaño et al., 2022; Kubayi, 2022) and, later, with the FIFA Women’s World Cup 2023 (Branquinho et al., 2024; Bradley, 2025a, 2025b; Iván-Baragaño et al., 2025; Oliva-Lozano et al., 2025).

Currently, Artificial Intelligence, and Machine Learning in particular, have become topics of interest for researchers and practitioners (Nassis et al., 2023; Rico-González et al., 2023), who have conducted studies with various objectives, such as establishing differences between men’s and women’s football (Pappalardo et al., 2021), predicting injury risk (Robles-Palazón et al., 2021), or the probability of success of different types of actions, such as entries into the penalty area (Iván-Baragaño et al., 2021; Stival et al., 2023) or shots during set-piece situations (Maneiro et al., 2019). In all of these studies, different regression and/or classification models were trained with the aim of predicting outcomes or future behaviors.

More recently, other studies have attempted to apply more complex strategies, materialized in the use of various techniques based on deep neural networks. Among the different examples of the use and application of Artificial Intelligence in the analysis of high-performance football, the article by AlMulla et al. (2023) trained a deep neural network model (Gated Recurrent Unit) to predict the outcomes of football matches in the Qatari league over 10 consecutive seasons, using data from data providers. Similarly, Wang et al. (2024) trained and evaluated a generative AI model based on deep learning and graph methods, which allowed the generation of execution proposals for set-piece actions. This was part of an unusual collaboration between Google DeepMind and Liverpool FC. Despite this, and in agreement with Claudino et al. (2019) the synergy that Artificial Intelligence needs to create alongside football still requires further development in the coming years.

This gap is even more pronounced in the case of women’s football, with scarce scientific evidence where AI or ML has been applied to female samples. In this regard, some authors have sought to understand the differences between men’s and women’s football (Pappalardo et al., 2021) using supervised ML techniques and applying explainability methods such as SHAP values (Lundberg and Lee, 2017). On the other hand, other studies have conducted analyses of offensive play using supervised techniques such as binary logistic regression (Iván-Baragaño et al., 2022), multinomial logistic regression (Casal et al., 2023), or decision trees (Maneiro et al., 2019). Additionally, some authors (Shen et al., 2024) have proposed models focused on convolutional neural networks and computer vision to determine offensive positioning in women’s football, using images extracted from UEFA Women’s Champions League matches.

In any case, and as a common aspect of studies conducted using supervised machine learning classification techniques, most studies have been carried out using methods characterized by high intrinsic explainability (such as decision trees or logistic regression), but often with moderate performance. In this context, there is a need to improve the performance of predictive models applied to a chaotic and non-linear reality like football, without sacrificing interpretability, to ensure the application of these studies’ results to training and competition.

For the reasons mentioned above, the objective of this study was twofold. First, it aimed to create two binary classification models that would allow the prediction of the outcome of ball possessions in elite women’s football (i.e., whether the possessions end in a Goal or a Shot). Additionally, once these models were trained, the SHAP library was implemented to identify the technical-tactical performance indicators that had the greatest influence on the model.

## 2 Materials and methods

### 2.1 Design and participants

The study was framed within the systematic observational methodology proposed by Anguera (1979) employing a nomothetic design, as multiple units of analysis were examined, represented by each participating team; it featured punctual inter-sessional tracking due to the temporal association between the actions analyzed within a single match; and it was multidimensional, as the observation instrument addressed the dimensions of identification, initiation, development, and outcome of ball possessions (Anguera et al., 2011).

All ball possessions during the final phase (from the Round of 16 onwards) of the FIFA Women’s World Cup 2023 were analyzed, provided they met the following inclusion criteria: (i) a minimum duration of 4 s, and (ii) the possession must involve two consecutive touches of the ball, a pass, or a shot (Almeida et al., 2014).

### 2.2 Observation and recording instrument

The observation instrument was created by a panel of experts, including three researchers with over 30 years of experience in observational methodology and can be consulted in Table 1. It comprised 18 criteria, and 51 categories. The analyzed criteria were organized in 4 dimensions corresponding to identification, start, development and outcome of the ball possession. The recording instrument used for this study was LINCE PLUS (Soto-Fernández et al., 2021).

**Table 1:** Observational instrument: criteria, categories, and operational definition.

Criteria	Categories	Operational definition
Observe team	Teams analyzed	The team that executed the ball possession
Match outcome	Win	The team observed won the match
	Lose	The team observed lost the match
	Draw	The team observed draw the match
Time	1Q	Possession starts between the start of the game and minute 1
	2Q	Possession starts between minute 16 and minute 30
	3Q	Possession starts between minute 31 and the end of the first half
	4Q	Possession starts between the start of the second half and minute 60
	5Q	Possession starts between minute 61 and minute 75
	6Q	Possession starts between minute 76 and the end of the game
Match status	Winning	The team observed is winning when the action starts
	Drawing	The teams are level when the action starts
	Losing	The team observed is losing when the action starts
Start form	Set Play	Possession begins after a regulatory interruption of the game.
	Transition	Possession begins without a regulatory interruption.
Start zone (length)	Defensive	Possession begins in the defensive area of the pitch
	Predefensive	Possession begins in the predefensive area of the pitch
	Middle	Possession begins in the middle area of the pitch
	Preoffensive	Possession begins in the preoffensive area of the pitch
	Offensive	Possession begins in the offensive area of the pitch
Start zone (width)	Left	Possession starts from the left wing
	Central	Possession starts from the center
	Right	Possession starts from the right wing
Defensive organization	Organized	The opposing team is defensively organized
	Circumstantial	The opposing team is defensively disorganized
Defensive positioning	Low	Opponents positioning is at the back at the start of the action
	Medium	Opponents positioning is midfield at the start of the action
	Advanced	Opponents positioning is forward at the start of the action
Interaction context	MM	Midfield zone vs. midfield zone
	RA	Rear zone vs. forward zone
	RM	Rear zone vs. midfield zone
	A0	Forward zone vs. goalkeeper
	AA	Forward zone vs. forward zone
	AM	Forward zone vs. midfield
	AR	Forward zone vs. rear zone
	MA	Midfield zone vs. forward zone
	MR	Midfield zone vs. rear zone
	PA	Goalkeeper vs. forward zone
Offensive intention	Keep	The team observed tries to maintain possession of the ball
	Progress	The team observed tries to progress towards the rival goal
Defensive intention	No pressure	The opposing team shows an intention to defend their goal
	Pressure	The opposing team shows an intention to recover the ball
MD (seconds)		Time of possession in own half (in seconds)
MO (seconds)		Time of possession in opponent’s half (in seconds)
Possession time		Total time of possession
Passes		Number of passes
Possession zone	MD	Most possession in own half
	MO	Most possession in opponent’s half
Possession outcome	Goal	The possession ends with a goal
	Shot	The possession ends with a shot
	Sent to area	The possession ends with a ball into the penalty area
	No success	The possession ends with no success.

### 2.3 Procedure and reliability

Prior to conducting the recording, the observers were trained and familiarized with the observation instrument over 4 sessions, following the procedure proposed by Losada and Manolov (2015). The reliability of the observation instrument was verified through the calculation of Cohen’s (1960) Kappa coefficient for both intra- and inter-observer reliability among the study’s authors. The average obtained was 0.869 (range: 0.729–0.979), which is considered excellent (Landis and Koch, 1977), based on the average of all criteria and observations made on 258 records corresponding to two matches.

### 2.4 Data cleaning and preprocessing

**Figure 1:**
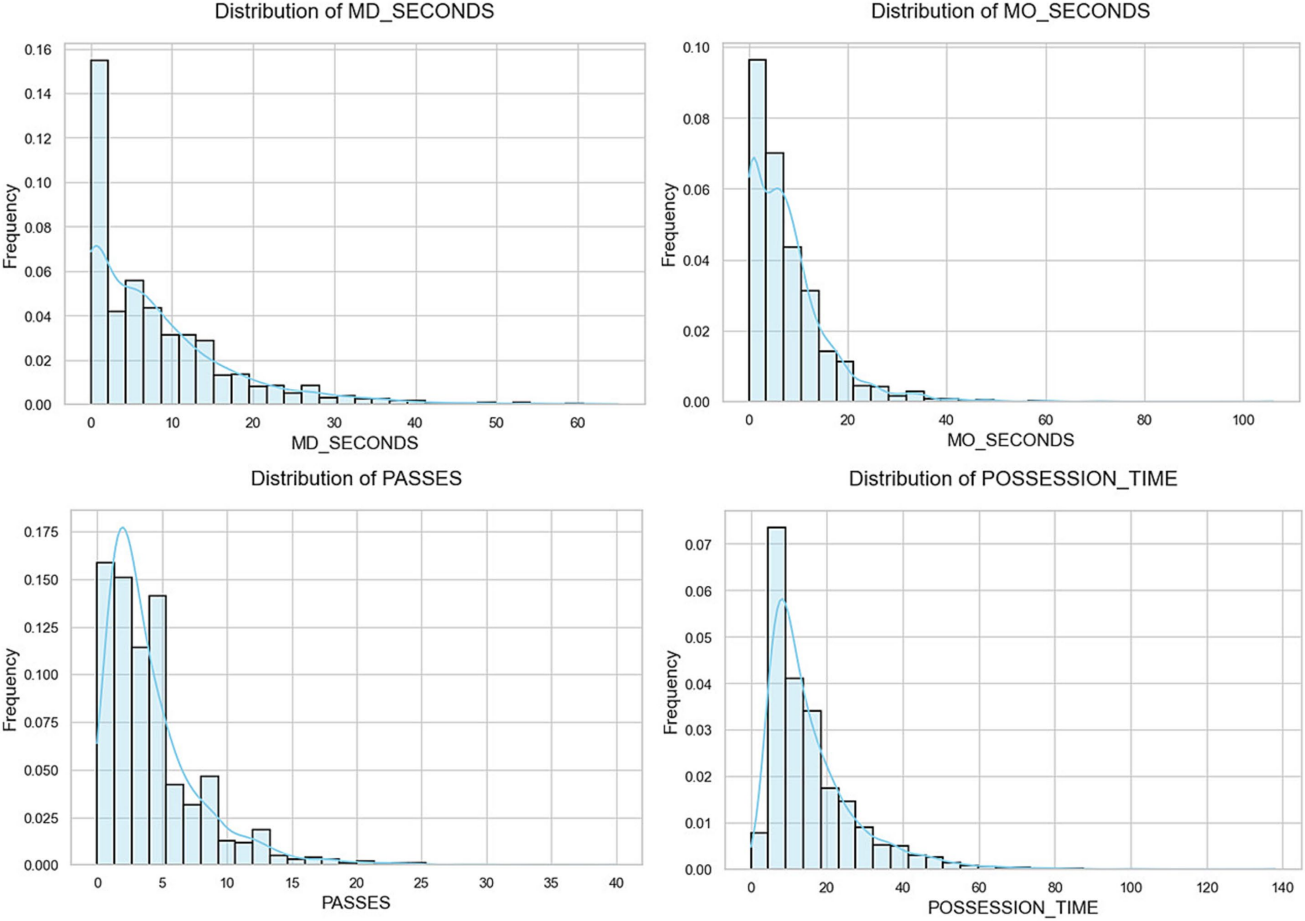
Initial distribution of quantitative variables.

Once the data matrix was obtained, consisting of 2,346 ball possession records, the following cleaning and preprocessing tasks were performed using the Scikit-Learn library (Pedregosa et al., 2011): (i) Checking for null values (none were found), (ii) Mapping the Possession Outcome variable into two binary recodings (Recoding 1: Success = Goal or Shot, No Success = Rest of the possessions & Recoding 2: Success = Goal, No Success = Rest of the possessions), (iii) Scaling of quantitative variables using the MinMaxScaler technique due to the skewness of the distribution (Figure 1), (iv) Applying OneHotEncoding to categorical variables.

**Figure 2:**
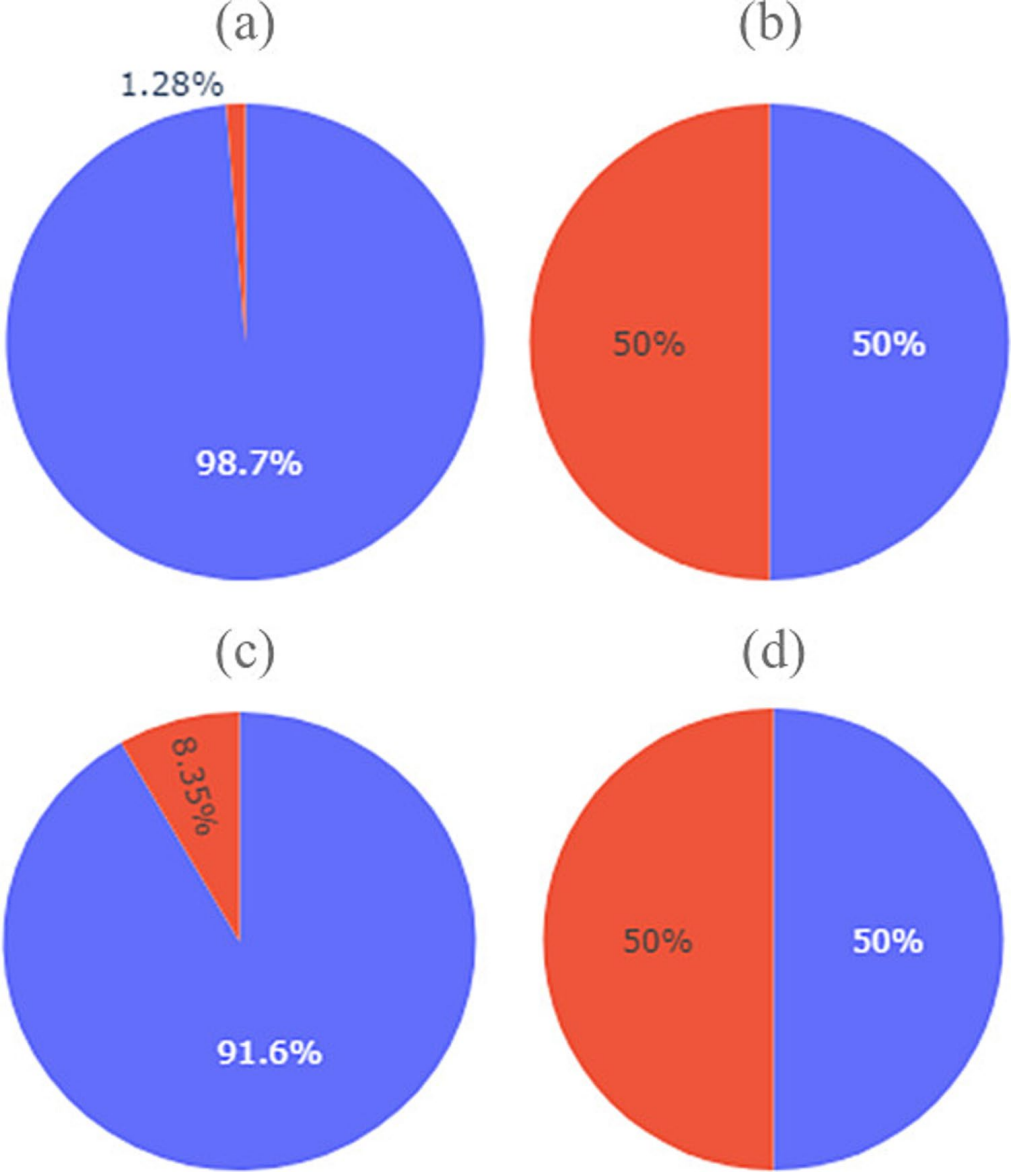
Initial distribution of success and no success classes for the two recodings before and after oversampling. **(A)** Percentage of goals in the original dataset, **(B)** percentage of goals in the resampled dataset, **(C)** percentage of goals or shots in the original dataset, **(D)** percentage of goals or shots in the resampled dataset.

When the dataset was preprocessed, an oversampling process was performed on the unbalanced class in both recodings (Success) using the Imbalanced Learn library (Lemaitre et al., 2017) which adjusted the classes to 50%. Figure 2 presents the percentage of positive cases for the target variable, considering success as a goal (Figures 2A,B) and as both a goal and a shot (Figures 2C,D). The oversampling process was carried out using SMOTE, due to its performance in model training in other studies (Last et al., 2017).

### 2.5 Data analysis

Once the datasets were resampled, the supervised machine learning models were trained using the Random Forest and XGBoost techniques, both implemented in the Scikit-Learn (Pedregosa et al., 2011) and XGBoost (Chen and Guestrin, 2016) libraries, respectively. The selection of these two algorithms is justified in this work to evaluate the classification capacity of different model combinations. In this context, the Random Forest model is considered one of the most powerful Bagging techniques, while XGBoost is classified within the Boosting techniques. The search for the best model was conducted through a cross-validation procedure using 5 folds on the training sample, which consisted of 80% of the total dataset. A grid search was performed using the following combination of hyperparameters:

- Random Forest Technique: (i) n_stimators (200, 300), (ii) max_depth (None, 10, 20, 30), (iii) min_samples_split (2, 5, 10), (iv) min_samples_leaf (1, 2, 4), and (v) Bootstrap (True, False)
- XGBoost: (i) n_stimators (200, 300), (ii) max_depth (3, 6, 9), (iii) learning_rate (0.01, 0.1, 0.2), (iv) subsample (0.6, 0.8, 1), and (v) subsample_by_tree (0.6, 0.8, 1)

Once the best model was obtained, it was trained on the resampled dataset, and its performance was evaluated on both the resampled test set and the original test set. All the steps carried out are published in the following repository (https://doi.org/10.6084/m9.figshare.27109405) and the dataset is available at the following link (https://doi.org/10.6084/m9.figshare.27109414).

## 3 Results

For both recoding 1 and recoding 2, the Random Forest algorithm demonstrated higher performance compared to the XGBoost algorithm. The combination of hyperparameters that provided the best performance for recoding 1 (Goal or Shot) was Random Forest: (i) n_estimators = 200, (ii) max_depth = None, (iii) min_samples_split = 5, (iv) min_samples_leaf = 1, Bootstrap = False. Similarly, for recoding 2 (Goal), the best performance was achieved with the following Random Forest combination: (i) n_estimators = 300, (ii) max_depth = None, (iii) min_samples_split = 5, (iv) min_samples_leaf = 1, Bootstrap = False.

### 3.1 Results of the classification models

**Figure 3:**
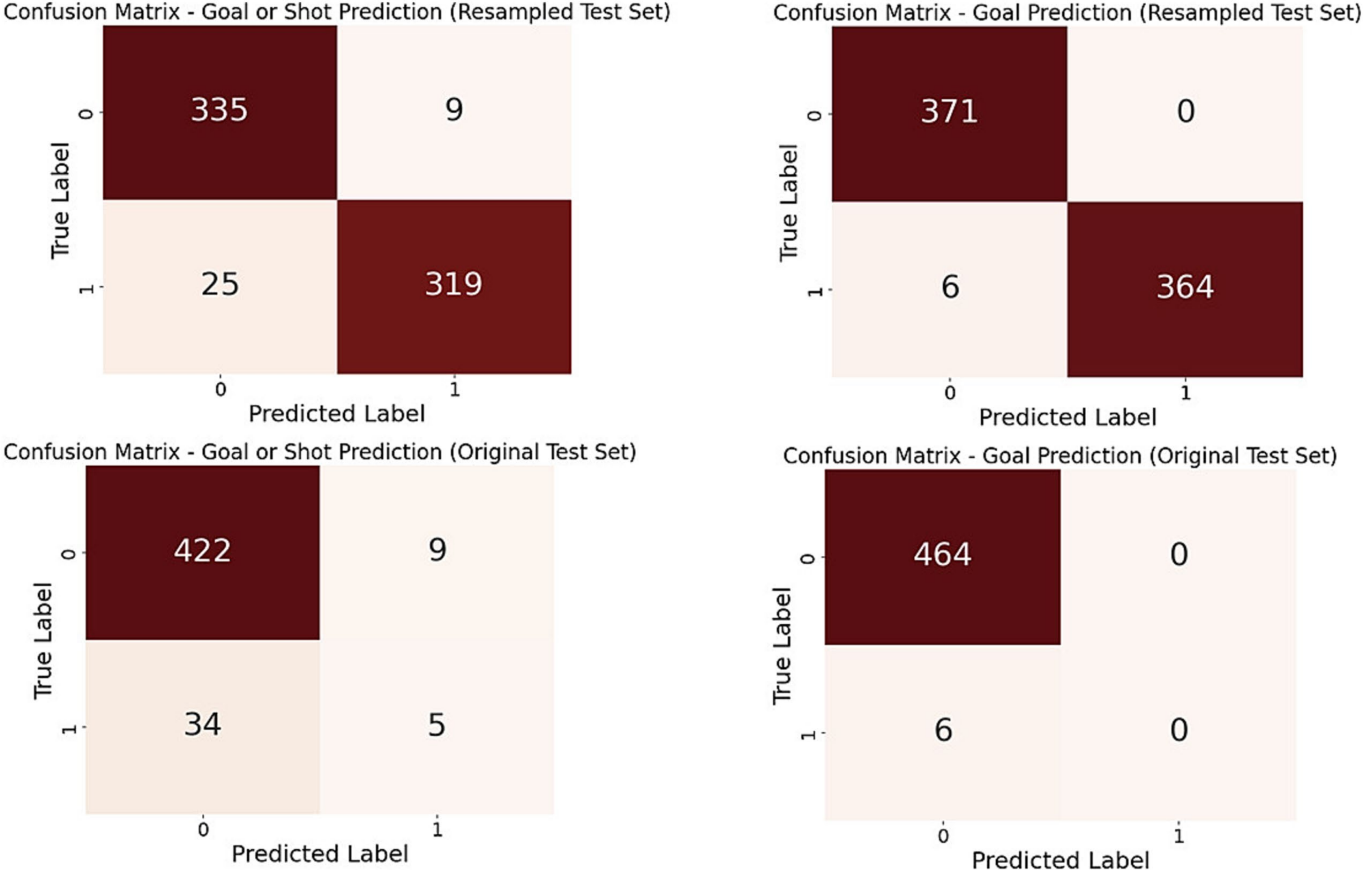
Confusion matrix of the model on the resampled and original test set (recoding 1 and 2).

The results of the classification models are presented in the form of a confusion matrix in Figure 3. Additionally, a summary of the main evaluation metrics is provided in Table 2. Overall, the models demonstrated excellent performance on the resampled test sets (recall = 0.93 and 0.98 for the first and second recoding, respectively). However, on the original test sets, the model was unable to generalize, showing an incomplete ability to predict the “Goal” outcome, with a recall of 0.

**Table 2:** Summary report of the models trained.

	Goal or shot prediction		Goal prediction	
	Resampled test set	Original test set	Resampled test set	Original test set
Random Forest model				
Accuracy	0.95	0.95	0.99	0.99
Recall	0.93	0.13	0.98	0
Specifity	0.97	0.98	0.99	1
XGBoost model				
Accuracy	0.95	0.90	0.99	0.99
Recall	0.92	0.10	1	0
Specifity	0.97	0.97	0.98	1

### 3.2 Influence of predictor variables on the model output

**Figure 4:**
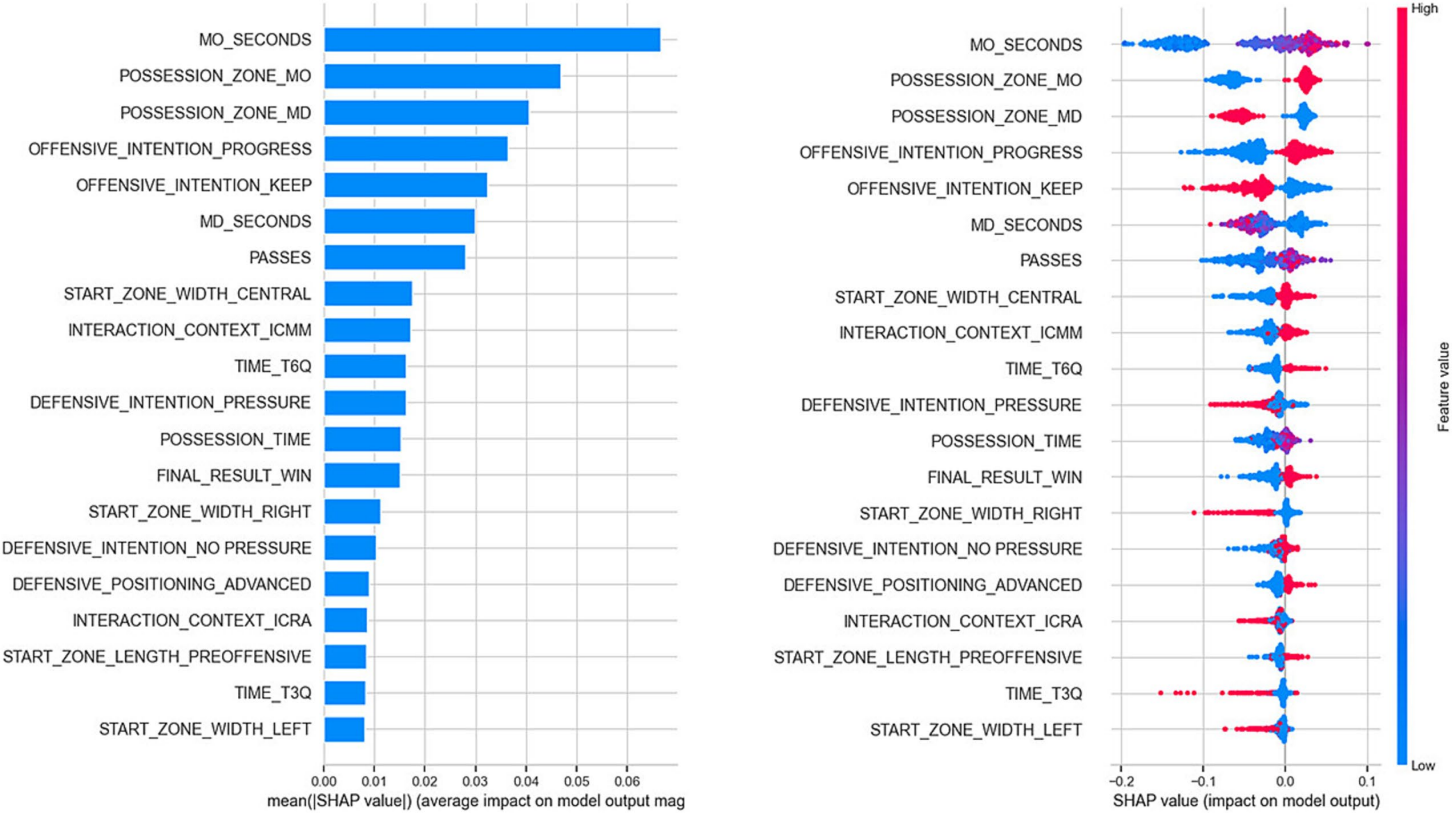
Influence of predictor variables on the model output (Success = Shot or Goal). In the left figure, the overall influence of the predictor variables is presented. In the right figure, the influence is shown based on the value of the predictor variable: pink colors indicate high values for the predictor variable, and blue colors indicate low values. For example, in the case of the first variable (MO_seconds), the blue colors are located to the left of the X-axis (below 0), indicating that when the variable has low values (short possession duration in the opponent’s half), the model decreases the likelihood of predicting the positive class (e.g., Goal or Shot). Lastly, for dichotomous variables (e.g., Offensive_intention_progress), the pink colors indicate the positive class of that variable (i.e., if there was an initial offensive intention to progress, then it is more likely that the model will predict the positive class for the target variable).

Figure 4 shows the influence of predictor variables on the model output for recoding 1 (Success = Goal or Shot). It was observed that the variable with the greatest influence was the duration of the attack in the opponent’s half, with higher values of this variable increasing the likelihood of a positive model output. Next, the variables with the most significant influence were the Possession Zone (dichotomous variables), confirming previous findings. Similarly, an initial offensive intention to progress increased the probability of a positive model output.

For the variable Passes, the color distribution observed on the X-axis indicated that possessions with mid-range values (purple colors located towards the right of the X-axis) increased the likelihood of obtaining a positive output. Lastly, the starting lane of possessions also had an influence: while possessions that began in the central lane increased the probability of a positive model result, those that started on the left and right lanes had a negative influence.

**Figure 5:**
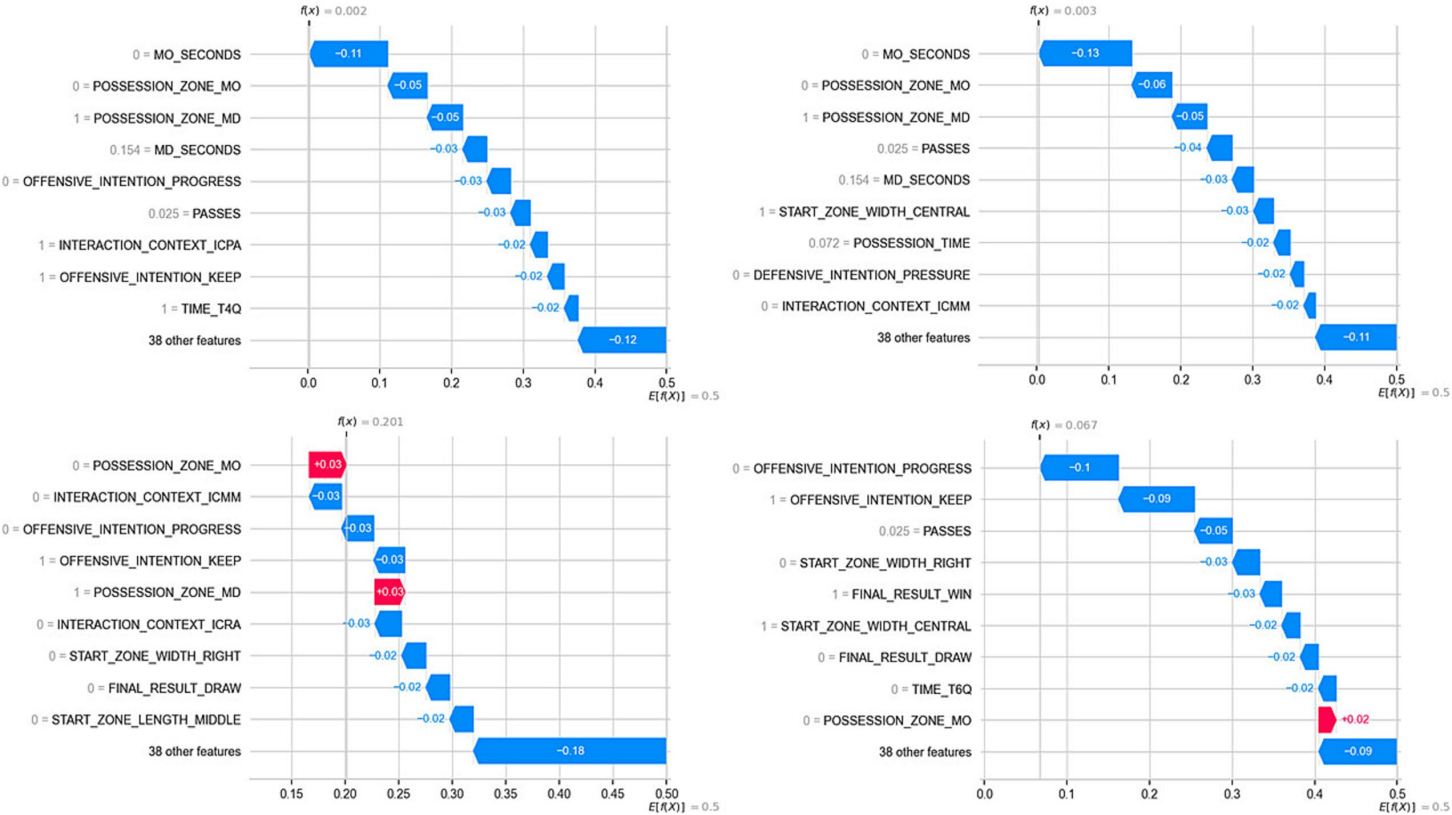
Influence of the features recorded in 4 random cases from the dataset on the model’s output. Pink colors indicate an increase in the probability that the model’s output will be the positive class of the target variable.

In Figure 5, the observed influence in 4 random cases from the original dataset is presented for each of the features recorded in those elements, which allows us to gain an individual understanding of the influence of these variables on the specific actions analyzed.

**Figure 6:**
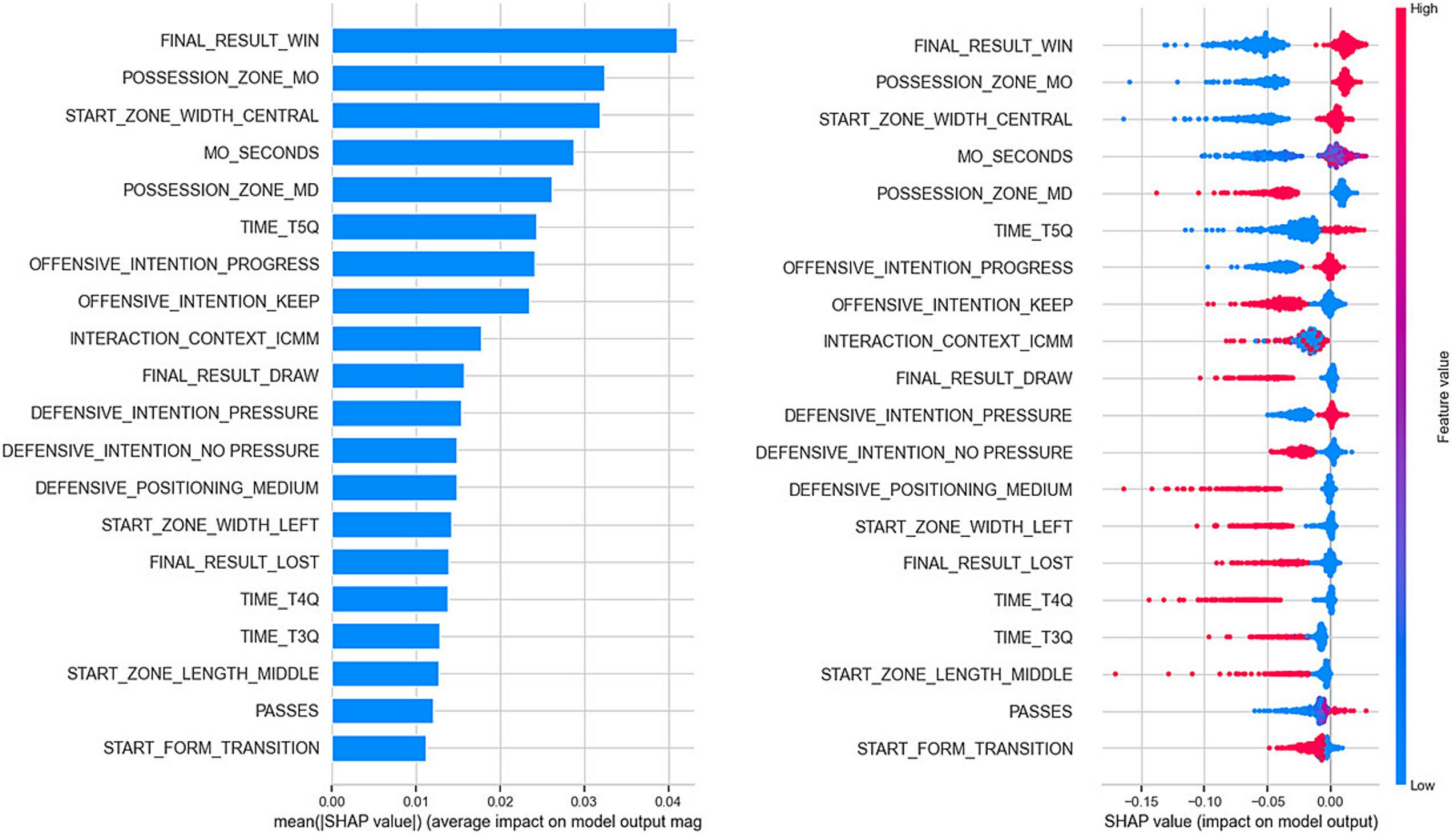
Influence of predictor variables on the model output (Success = Goal).

**Figure 7:**
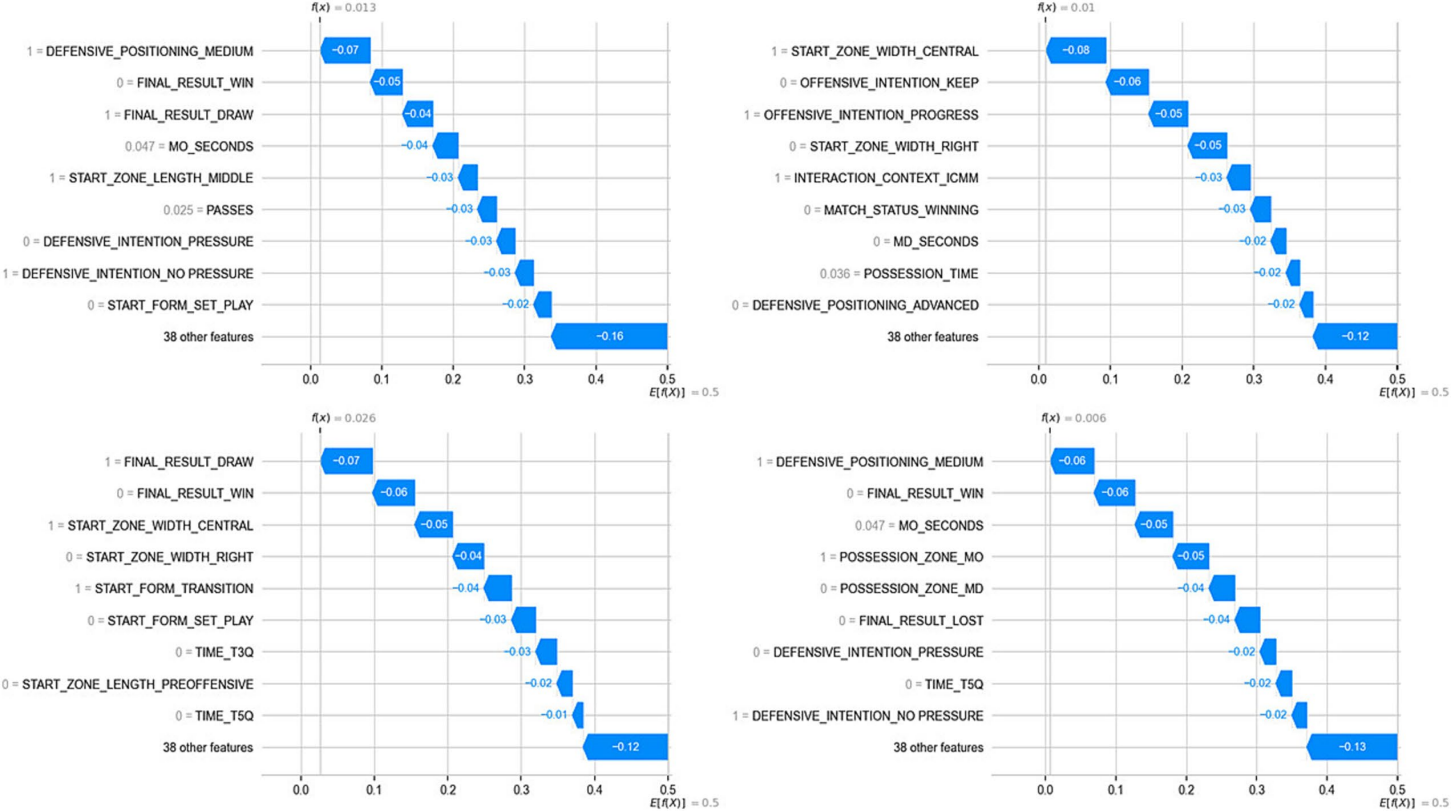
Influence of recorded features in 4 random cases from the dataset on the model’s output.

Lastly, Figure 6 presents the overall influence of the predictor variables on the model’s output for recoding 2 (Success = Goal). In this model, the variable with the greatest influence was Match Outcome (Winner), followed by the variables Possession Zone (MO), Start Zone Width (Central), Possession Time in Opponent’s Field, and Time (5Q). In this figure, an evident issue of collinearity between the target variable and the most influential variable in the model (Match Outcome = Winner) was observed, which may be the cause of the model’s poor performance on the test set. Additionally, Figure 7 presents the local influence of the recorded features in four specific cases from the analyzed dataset, aiming to show how the probabilities of success are modified based on the recorded variables.

## 4 Discussion

The objective of this study was, first, to create two binary classification models that could predict the outcome of ball possessions in elite women’s football. Additionally, once the models were trained, the aim was to identify the technical-tactical indicators associated with a higher probability of achieving a goal or a shot during ball possessions. To achieve these objectives, a mapping of the Possession Outcome variable was performed based on the degree of success (Goal or Shot). Following this, oversampling of the imbalanced class was conducted.

Previous studies have employed similar procedures with the aim of predicting the outcome of ball possessions in women’s football. However, in most of these studies, success was defined as reaching the penalty area, reaching the final third, or, more generally, the creation of Goal Scoring Opportunities (Scanlan et al., 2020; Kubayi, 2022; Mitrotasios et al., 2022; Mesquita et al., 2023). This aspect is crucial when training a classification model, as approximately one in four (25%) ball possessions in women’s football ends with a move into the final third or the opponent’s penalty area (Iván-Baragaño et al., 2021; Casal et al., 2023), allowing a balance between correctly classified positive and negative cases. In contrast, in this study, the dataset showed a percentage of positive cases of 1.35 and 8.28%, respectively, which necessitated oversampling of the imbalanced classes to prevent the model from ignoring the minority class (Haller et al., 2023).

The classification models yielded excellent results on the resampled datasets, with recall and specificity exceeding 93% in both models. However, their performance on the original datasets was poor. When predicting shots or goals, the model had a recall of 13%, and in the case of goal prediction, the model did not predict any positive outcomes. These results highlight the difficulty of predicting infrequent events in football, such as shots and goals, and underscore the need for incorporating a larger number of predictor variables, as well as further tuning the hyperparameters during model training. Similarly, as seen in injury prediction, where different studies have shown recalls between 10 and 15% (Haller et al., 2023; Majumdar et al., 2024), the holistic nature of the sport contributes to the challenge of accurately predicting such events.

In relation to the SHAP technique (Lundberg and Lee, 2017) applied in this study, it was found that a large number of indicators associated with ball possessions contributed to increasing the probability of a favorable outcome for the executing team. The performance indicators associated with successful ball possessions in elite women’s football observed in this work largely align with previous studies on this topic. In this regard, Maneiro et al. (2022) demonstrated that developing ball possessions in the opponent’s half increased the likelihood of the possessions ending with a delivery into the penalty area. Similarly, the offensive tactical intent once ball possession was initiated, or the number of passes made in the offensive sequence, were variables that significantly altered the outcome of ball possessions in women’s football (Scanlan et al., 2020; Iván-Baragaño et al., 2021; Casal et al., 2023).

However, considering that the level of success analyzed in this study was higher than in previous studies, this work also demonstrated the existence of variables that had not previously shown a multivariate influence on the outcome of ball possessions. For example, in the study by Iván-Baragaño et al. (2022), it was observed that the current match score had an influence on the development and outcome of ball possessions. Thus, it is interesting to note that, while success in delivering the ball into the penalty area can be influenced by the flow of the game, when it comes to taking a shot or scoring a goal, this variable does not have sufficient influence. This insight may have significant implications for the sport, as it could suggest that when teams are losing, they tend to deliver the ball into the penalty area more often but are less successful in converting these deliveries into shots or goals.

Similarly, it is interesting to analyze the influence of the variables Time (5Q and 6Q) and Start Zone Width (Central). According to the SHAP values generated for these variables, the following insights can be drawn. When predicting a shot, the likelihood increases if the possession occurs in the last 15 min of the match (6Q). However, when analyzing the SHAP values for the positive outcome “Goal,” the probability increases between the 60th and 75th minutes of the match. This contradicts the findings from the 1999, 2003, and 2007 World Cups, where a higher number of goals were observed in the final 15 min of the match (Armatas et al., 2007), as well as the results from the most recent Women’s Euro 2022 (Sanmiguel-Codina et al., 2025).

Additionally, the observation that starting an attack in the central lane (Start Zone Width = Central) increases the probability of success had not been noted in previous studies (Scanlan et al., 2020; Iván-Baragaño et al., 2021; Maneiro et al., 2022). This may suggest that while starting attacks from wide areas may facilitate successful entries into the penalty area, shots and goals are more likely to result from attacks initiated in central zones.

This study presents several limitations that should be addressed in future research. First, while the classification models achieved excellent performance on oversampled datasets using the SMOTE technique, their ability to detect true positives in the original dataset was notably poor. From a football perspective, this suggests that the actions leading to dangerous situations may follow highly specific patterns that generic classification models, such as Random Forest, are unable to effectively capture. In this context, future studies might benefit from the implementation of advanced statistical techniques like T-Patterns, which have proven effective in identifying offensive patterns and sequences in other sports (Pic and Jonsson, 2021; Pic et al., 2021). Additionally, exploring alternative tools to mitigate overfitting during model training is essential. Expanding the dataset by analyzing additional championships could also enhance the robustness of the identified patterns related to goal scoring. Furthermore, the inclusion of certain predictor variables, such as Match Outcome, was found to influence model performance, not due to their predictive capability, but because of their retrospective causal relationship (e.g., the winning team scored more goals). This introduces data leakage during model training. Consequently, future research should consider excluding such variables from the training process to ensure more reliable and generalizable results.

## 5 Conclusion

The models trained and tested in this study showed excellent performance on the resampled datasets using the SMOTE technique (Last et al., 2017). However, when these models were evaluated on the original dataset, their performance was low or non-existent. In the case of predicting Goals or Shots, the model achieved a recall of 13%, which slightly increased the relative frequency of the positive class but fell far short of an acceptable performance. For goal prediction, the model was unable to output the positive class at all. Based on this, it can be stated that such events in elite women’s football possess very specific characteristics and patterns that cannot be clearly defined or that, at least, involve variables not analyzed in this study.

On the other hand, the SHAP explainability techniques applied in this study allowed for the identification of various variables associated with the achievement of goals and shots. Some of these variables showed similarities to previous studies, where success was categorized as entries into the penalty area or similar metrics. However, other variables such as start zone width, timing, or defensive intent had a significant influence on the model when analyzing a higher degree of success, enabling a tactical understanding of how these types of actions occur.

## Data Availability

The datasets presented in this study can be found in online repositories. The names of the repository/repositories and accession number(s) can be found in the article/supplementary material.
